# Chlorogenic Acid Improves Quality of Chilled Ram Sperm by Mitigating Oxidative Stress

**DOI:** 10.3390/ani12020163

**Published:** 2022-01-11

**Authors:** Yanhu Wang, Liuming Zhang, Tariq Sohail, Yan Kang, Xiaomei Sun, Yongjun Li

**Affiliations:** College of Animal Science and Technology, Yangzhou University, Yangzhou 225009, China; WYH18805274475@163.com (Y.W.); 18352767281@163.com (L.Z.); Drtariqsohail34@yahoo.com (T.S.); ky2583141771@163.com (Y.K.)

**Keywords:** chlorogenic acid, Hu ram sperm, chilling storage, oxidative stress

## Abstract

**Simple Summary:**

Sheep sperm is extremely sensitive to reactive oxygen species (ROS) and can produce a large amount of ROS during chilling storage, leading to a decline in semen quality. Adding antioxidants is an important method to improve semen quality. Chlorogenic acid (CGA) is a kind of plant extract with an antioxidant capacity, which can effectively eliminate free radicals and improve the antioxidant capacity of semen. However, its role in the chilling storage of Hu ram semen is not clear. Therefore, CGA with different concentrations was added to chilling storage extender to investigate its effect on chilled ram sperm. The results of this study revealed that CGA with proper concentration had a positive effect on chilled Hu ram sperm and 0.8 mg/mL CGA had the best effect.

**Abstract:**

The purpose of this study was to investigate whether the addition of chlorogenic acid (CGA) to a sheep semen extender could improve the quality of chilled sheep sperm. Ejaculates (*n* = 80) were collected from five Hu rams with an artificial vagina. The ejaculates were mixed and divided into five equal parts, diluted with a CGA-free Tris–egg yolk extender (control), or supplemented with 0.2, 0.4, 0.8, and 1.2 mg/mL. The sperm kinematic parameters (viability, progressive motility), functional integrity of plasma membrane and acrosome, adenosine triphosphate (ATP) concentration and antioxidant parameters (Catalase (CAT), Superoxide dismutase (SOD) activity, total antioxidant capacity (T-AOC), ROS level and Malondialdehyde (MDA) content) were evaluated during storage of the semen. The results indicated that: PM, plasmatic membrane integrity and acrosomal integrity in 0.8 mg/mL CGA were higher (*p* < 0.05) from day 1 to 5. The ROS level in CGA groups was lower than the control (*p* < 0.05). CAT, SOD, ATP, and T-AOC were highest at 0.8 mg/mL concentration within 1 to 5 days. The above results indicated that the right concentration of CGA improved the quality of Hu ram sperm during chilling storage.

## 1. Introduction

Hu sheep is a valuable sheep breed in China, with high-quality meat and skin. It has the advantages of early sexual maturity, fast growth, four season estrus, and two offspring a year [1]. It can effectively make up for the defects of sheep fattening in autumn and winter, and fully meet people’s demand for mutton if breeding on a large scale. Natural mating is influenced by factors such as geography and the spread of reproductive diseases, which inhibits the reproductive potential of dominant male varieties. Therefore, artificial insemination (AI) technology is very important in large-scale breeding farms. The quality of semen storage is key to the effect of artificial insemination. When stored at room temperature, sperm motility decreases sharply in a short time and cannot meet the needs of long-distance transportation [2]. Otherwise, cryopreservation of semen causes serious damage to sperm, which decreases the ability of sperm fertilization and affects the potential fertility of artificial insemination [3]. The preservation of semen at low temperature can effectively slow down the metabolism of sperm and prolong its survival time [4], which is of great significance for overcoming geographical limitations and the cultivation of excellent animals’ genetic characteristics. Liu et al. found that the blastocyst rate of cryopreserved sheep sperm after fertilization was low (29.12 ± 3.01) [5]. Studies revealed that the rate of ewes fertilized with frozen-thawed sheep sperm was 4% [6]. It was reported that the pregnancy rate of ewes fertilized with frozen semen (preserved at 15 C for 6 h) was 52% [7]. Therefore, chilling storage technology for semen has been widely used by sheep farmers and breeding enterprises.

Sperm are susceptible to external factors such as temperature, pH, osmotic pressure, and internal factors such as their own metabolic products [8,9] during storage. Excessive ROS can combine with PUFAs in sperm plasma membrane, causing lipid peroxidation and finally destroying the membrane structure and function decline of sperm plasma [10]. In addition, lipid peroxidation can interact with related proteins in mitochondrial electron transport chain, which leads to the production of lipid aldehydes, and then produces more ROS, eventually damaging DNA in the sperm nucleus and leading to sperm death [10,11]. The lipid composition of the sheep sperm membrane is different from that of somatic cells, and it is rich in PUFAs, which makes sperm susceptible to physical, chemical, and oxidative damage caused by the accumulation of ROS during storage [4,12]. Sperm cells are deficient in endogenous antioxidants, which leads to the decline in integrity of the sperm plasma membrane [5]. Therefore, adding exogenous antioxidant substances has become one of the effective methods to improve the quality of semen preservation and pregnancy rate. Studies have shown that bioactive peptides isolated from natural herbs or mammalian organs effectively eliminated the production and accumulation of ROS [5]. At present, it has been reported that vitamin E [13], CoQ10 [14], reduced glutathione (GSH) [15,16] astaxanthin [17], and melatonin [18,19] have positive effects on sperm of sheep, humans, dogs, boars, and rats. Most antioxidants, however, tend to have both positive and negative effects on sperm: the appropriate concentrations of antioxidants protect sperm, whereas a high concentration has a toxic effect [20].

Chlorogenic acid (CGA), a polyphenol compound with antibacterial, antioxidant, and anti-inflammatory properties, has been applied to the food, cosmetics, and pharmaceutical industries. In addition, it also has the ability to eliminate free radicals [21]. Recent research findings revealed that CGA significantly inhibits the expression and secretion of IL-8 mRNA in mouse intestinal epithelial Caco-2 cells caused by oxidative stress [22,23]. It has been reported that the sperm count of epididymis increased by 20% after 5 weeks of CGA administration in rats [24]. CGA improved the quality of cooled and frozen-thawed boar sperm [25,26,27]. Furthermore, CGA improved the antioxidant capacity of human sperm in vitro and during the frozen-thaw stage [28]. A variety of antioxidants have positive effects on chilled ram semen, such as argan oil [29], royal jelly [30], and Mito-TEMPO [31]; however, there are few reports on the study of CGA on chilled sheep semen. This study was designed to investigate the effects of CGA with the proper concentration on sperm quality and ability to attenuate oxidative stress of Hu sheep during chilling storage, which can provide basic references for semen reservation in Hu sheep.

## 2. Materials and Methods

### 2.1. Animals, Semen Collection and Processing Procedures

The five rams were kept in a facility at the Agriculture of Yangzhou University Agriculture. They are given straw, hay, and mixtures. All the procedures for animal treatment and sample collection were approved by the Ethical Committee of Experimental Animal of Yangzhou University, Jiangsu, China (license number: SYXK[Su]2017-0044). Ejaculates (*n* = 80) were collected from five rams by an artificial vagina. The semen samples were taken to the lab, and examined for volume, concentration, and viability. Only ejaculates with a volume ≥ 0.5 mL, concentration ≥ 2.0 × 10^9^/mL, and viability ≥ 0.8 were included in this study. After passing evaluation, fresh semen from five rams was mixed to reduce errors due to individual differences. The mixed semen was divided into 5 equal fractions and diluted with CGA without antioxidant and different concentrations; finally, the samples were stored at low temperature for subsequent tests.

### 2.2. Chemicals

Chlorogenic acid was purchased from Beijing Solarbio Science & Technology Co., Ltd. (Beijing, China). CGA (120 mg) was dissolved in DMSO (1 mL) to obtain CGA basic mother liquor. Unless otherwise specified, the rest of the chemicals were purchased from Sangon Biotech (Shanghai) Co., Ltd. (Shanghai China).

### 2.3. Semen Processing and Evaluation

Tris (3.07 g), fructose (2.00 g) and citric acid (1.64 g) were dissolved in 100 mL distilled water. After the basic extender (90 mL) was added with 10 mL of egg yolk, the mixture was fully stirred until completely dissolved, stored at 4 °C overnight, and centrifuged at 13,000× *g* for 15 min to collect the supernatant for later use. Each mixed semen sample was divided into five equal aliquots to diluted with 0.2, 0.4, 0.8 and 1.2 mg/mL CGA. Semen was diluted ten times with an extender containing Tris, and the final sperm concentration was adjusted at 100 × 10^6^/mL. The semen sample was stored at 4 °C in a refrigerator. Sperm kinematic parameters, plasmatic membrane integrity and acrosome integrity were assessed every 48 h for 7 days. Total antioxidant capacity(T-AOC), Superoxide dismutase (SOD), Catalase (CAT) activity, adenosine triphosphate (ATP) and Malondialdehyde (MDA) content were determined at the 1st, 3rd, and 5th days. ROS level was evaluated at 5th day. 

### 2.4. Sperm Kinematic Parameters

Sperm kinematic parameters were evaluated using Mailang sperm automatic analysis system ver5.0 (Instrument number: ML-608JZ II, Mailang, Nanning, China), including viability, PM, Curvilinear velocity (VCL, µm/s), Straight line velocity (VSL, µm/s), Average path velocity (VAP, µm/s). Briefly, 20 µL of chilled semen sample was diluted five times with extender, and incubated at 37 °C for 2 min. A total of 10 µL of incubated semen sample was added to the sperm count plate for evaluation of sperm kinematic parameters.

### 2.5. Sperm Plasma Membrane Integrity Assessment

Sperm plasma membrane integrity was evaluated using a hypotonic swelling test (HOST). A 10 µL semen sample was mixed with 100 µL hypo-osmotic solution (consisting of 0.90 g fructose and 0.49 g sodium citrate dissolved in 100 mL ultrapure water), the mixture was incubated at 37 °C for 30 min. The percentage of tails coiled in 200 sperm was counted using a 400× phase contrast microscope.

### 2.6. Sperm Acrosomal Integrity Assessment

The integrity of sperm acrosomal was evaluated using coomassie brilliant blue G-250 staining. Briefly, a 50 µL semen sample was mixed with 1 mL 4% paraformaldehyde to fixed for 10 min, the mixture was centrifuged at 1500× *g* for 5 min to obtain precipitate sperm cells, and made a smear. The smear was stained using Coomassie brilliant blue G-250 dye solution (Coomassie brilliant blue G-250 (0.10 g) was dissolved in 95% ethanol (50 mL), then 85% phosphoric acid (100 mL) was added, and the volume of the final staining solution was adjusted to 1 L). The percentage of the acrosome stained blue in a total of 200 sperm was counted using a 1000× oil immersion.

### 2.7. Determination of ROS in Sperm

ROS level was evaluated using a ROS assay kit (Solarbio, Beijing). Briefly, 50 µL chilled semen sample was washed with 500 µL PBS, the semen sample was treated with 300 µL 10 µmol/L DCFH-DA working solution, and incubated at 37 °C for 30 min. The mixture was centrifuged at 1000× *g* for 10 min to obtain precipitated sperm cells, the precipitate was washed with PBS three times to remove DCFH-DA from the non-entering sperm. The level of ROS was expressed as absorbance at an excitation wavelength of 488 nm and an emission wavelength of 525 nm for a microporous multimodal detection system.

### 2.8. Evaluation of Sperm Protein Concentration

Sperm protein concentration was evaluated using Bradford protein concentration determination kit (Beyotime institute of Biotechnology, Shanghai). A 50 µL sample was centrifuged at 550× *g* for 10 min to remove the supernatant, the semen sample were resuspended in lysis buffer. The lysate was centrifuged at 12,000× *g* for 5 min at 4 °C to obtain the supernatant. G250 staining solution (detergent compatibility, 300 µL) was added to the sample (10 µL), the absorbance was measured at 595 nm, and the protein concentration was obtained according to the standard curve [32,33].

### 2.9. Evaluation of T-AOC of Seminal Plasma

T-AOC of seminal plasma was measured using Total Antioxidant Capacity Determination Kit (Nanjing Jian Cheng Institute of Biological Engineering, Nanjing). Simply put, the standard Trolox solution (10 mM) was diluted using distilled water to 0.1, 0.2, 0.4, 0.8, 1.0 mM to obtain a standard curve. A total of 0.1 mL semen sample was centrifuged at 1500× *g* for 10 min to obtain supernatant. A total of 10 µL of supernatant was added into each well of the 96-well plate, and the corresponding reagents were added according to kit instructions. The mixture reacted at room temperature for 6 min, and the absorbance at 405 nm was obtained by a microporous multimode detection system. Since the extender contained egg yolk, a control only with extender was set to eliminate the effect of egg yolk. The results were calculated according to the standard curve.

### 2.10. Evaluation of CAT Activity in Seminal Plasma

CAT activity of seminal plasma was evaluated using Catalase Assay Kit (Nanjing Jian Cheng Institute of Biological Engineering, Nanjing). The basis for evaluating CAT activity is that ammonium molybdate could quickly terminate the reaction of catalase to decompose hydrogen peroxide, and H_2_O_2_ could react with ammonium molybdate to produce a pale yellow complex. A total of 0.2 mL diluted semen sample was centrifuged at 1500× *g* for 10 min to take the supernatant; the corresponding reagents were added in turn according to the kit instructions. Since the extender contained egg yolk, a control only with the extender was set to eliminate the effect of egg yolk. The absorbance of mixture was measured at 405 nm using a microporous multimode detection system.

### 2.11. Assessment of MDA Content in Sperm

Made a standard curve is based on the lipid oxidation (MDA) detection kit (Beyotime institute of Biotechnology, Shanghai). After resuspension, a 50 µL semen sample was lysed to fully release the MDA in the sperm. The lysate was centrifuged at 12,000× *g* at 4 °C for 5 min. After centrifugation, 0.1 mL sample was mixed with 0.2 mL MDA detection working solution, the mixture was treated with a boiling water bath for 15 min, cooled to room temperature, and centrifuged at 1000× *g* for 10 min. The absorbance of mixture was obtained at 532 nm using a microporous multimode detection system. The MDA content was obtained according to the standard curve, and the protein concentration was measured according to the procedure in Section 2.8. MDA content in sperm was presented in nm/mg protein.

### 2.12. Determination of SOD Activity in Sperm

A 50 µL semen sample was washed with PBS; 300 µL SOD liquid sample preparation was added to fully crack the cells, and the lysate was centrifuged at 12,000× *g* at 4 °C for 5 min to take the supernatant. In strict accordance with a SOD activity detection kit (Beyotime institute of Biotechnology, Shanghai) manual operation, the semen sample was incubated at 37 °C for 30 min, the absorbance of mixture at 450 nm was measured, and the protein concentration was measured as in Section 2.8. The results were expressed in unit/mg protein.

### 2.13. Determination of ATP Content

The semen sample was washed with PBS and centrifuged at 1500× *g* for 5 min to obtain precipitation cells, 500 µL double steaming hot water was added to the sample, then placed in a hot bath homogenate and broken. The mixture was treated in a boiling water bath for 10 min, and centrifuged at 4000× *g* for 5 min to take supernatant based on the instructions of ATP assay kit (Nanjing Jian Cheng Institute of Biological Engineering, Nanjing). The absorbance at 636 nm was obtained using a microporous multimode detection system, and the determination of protein concentration was shown in Section 2.8. The results were presented in µmol/mg protein.

### 2.14. Statistical Analysis

This study was replicated six times. The experimental data were analyzed by Statistical Package for the Social Sciences (SPSS, IBM, version 24.0). The Shapiro–Wilk tested normal distribution of data. All experimental results were presented as Mean ± SEM and compared using Duncan’s multiple range tests by one-way analysis of variance procedures. *p* < 0.05 indicated significant difference.

## 3. Results

### 3.1. Effects of CGA on Sperm Viability and PM

Viability and PM rate of 0.8 mg/mL and 1.2 mg/mL groups was higher than other groups (*p* < 0.05) on the 1st day. On the 3rd day, PM of 0.4 mg/mL and 0.8 mg/mL groups was higher than control group (*p* < 0.05). The viability and PM of all CGA groups were higher than control group (*p* < 0.05), the 0.8 mg/mL CGA group was highest from day 5 and 7. On the 3rd day, the VSL, VCL and VAP of all the CGA groups had no significant differences compared with the control group. Compared with the control group, the VAP of sperm in all CGA groups was higher (*p* < 0.05) on the 5th day. From day 5 to 7, the value of VCL and VAP in 0.8 mg/mL group was the highest (Table 1).

### 3.2. Effects of CGA on Sperm Plasma and Acrosomal Membrane Integrity

Compared with the control group, the sperm plasma membrane of all CGA supplementation groups was higher (*p* < 0.05) within 3 to 5 days. On the 7th day, the sperm plasma membrane integrity of 0.8 mg/mL group was the highest. On the 3rd day, the sperm acrosome integrity of 0.4 mg/mL and 0.8 mg/mL groups was higher than other groups (*p* < 0.05). On the 5th day, the sperm acrosome integrity in all CGA groups was higher than control group (*p* < 0.05), 0.8 mg/mL group was the highest (Table 2).

### 3.3. Effects of CGA Supplementation on Sperm ROS Content

**Figure 1:**
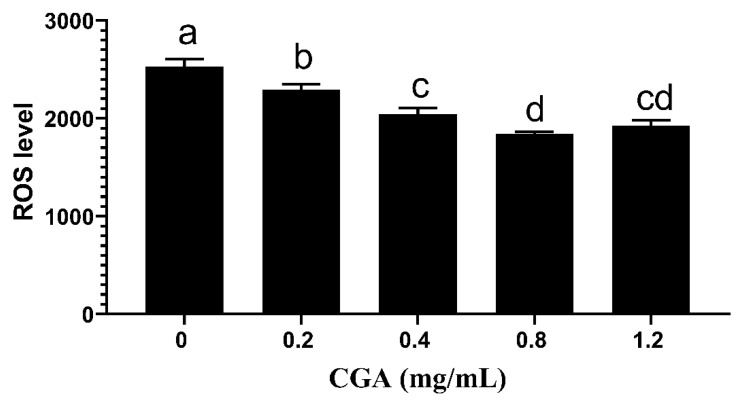
Effects of CGA with different concentrations on ROS level of chilled sperm on the fifth day. Note: Different letters above the column indicate significant difference, the same or containing the same letter indicate insignificant difference.

The ROS levels of CGA-treated groups decreased than the control group (*p* < 0.05), the value of 0.8 mg/mL group was lowest, and no significant difference was observed between 0.4 mg/mL and 1.2 mg/mL groups (Figure 1).

### 3.4. Effects of CGA on CAT Activity in Seminal Plasma

**Figure 2:**
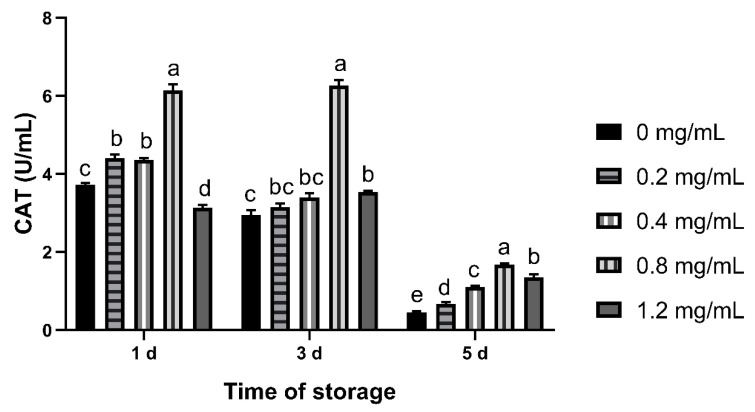
Effects of CGA with different concentrations on CAT activity of chilled ram semen. Note: Different letters above the column indicate significant difference, the same or containing the same letter indicate insignificant difference.

The CAT activity of 0.8 mg/mL group was highest from day 1 to 5 (*p* < 0.05). On the 5th day, the CAT activity of CGA supplementation groups was higher than the control group (*p* < 0.05, Figure 2).

### 3.5. Effects of CGA on T-AOC in Seminal Plasma

**Figure 3:**
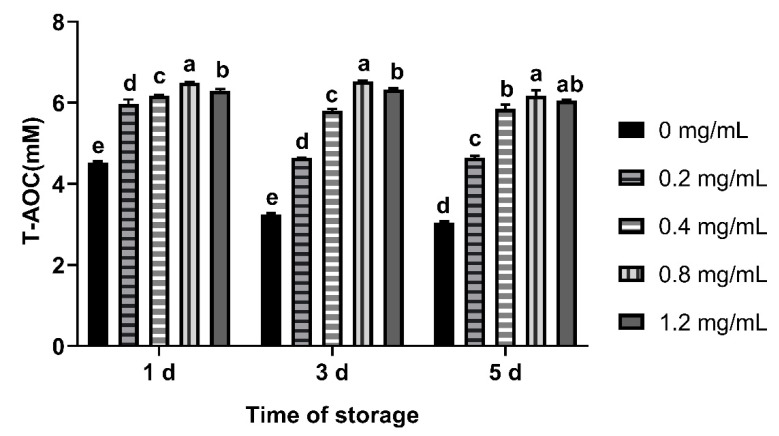
Effects of CGA with different concentrations on T-AOC of chilled ram semen. Note: Different letters above the column indicate significant difference, the same or containing the same letter indicate insignificant difference.

Compared with the control group, T-AOC of CGA added groups was higher (*p* < 0.05), 0.8 mg/mL group was the highest from day 1 to 5. No significant difference was observed between o.4 mg/mL and 1.2 mg/mL group on the fifth day (Figure 3).

### 3.6. Effects of CGA on MDA in Sperm

**Figure 4:**
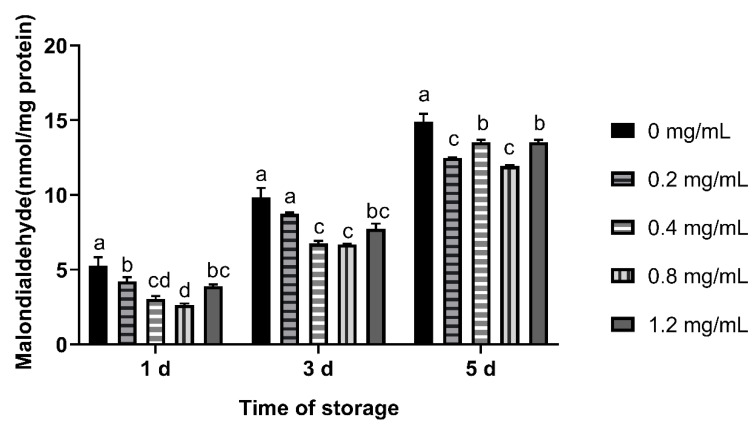
Effects of CGA with different concentrations on MDA content of chilled ram sperm. Note: Different letters above the column indicate significant difference, the same or containing the same letter indicate insignificant difference.

On the 1st day of semen preservation, the MDA content of all CGA added groups was lower than control group (*p* < 0.05), and that of 0.4 mg/mL and 0.8 mg/mL group was lower than 0.2 mg/mL group (*p* < 0.05). On the 3rd day, no significant difference was observed among 0.4~1.2 mg/mL groups. On the 5th day, MDA content in CGA added groups was lower than control group (*p* < 0.05, Figure 4).

### 3.7. Effects of CGA on SOD Activity in Sperm

**Figure 5:**
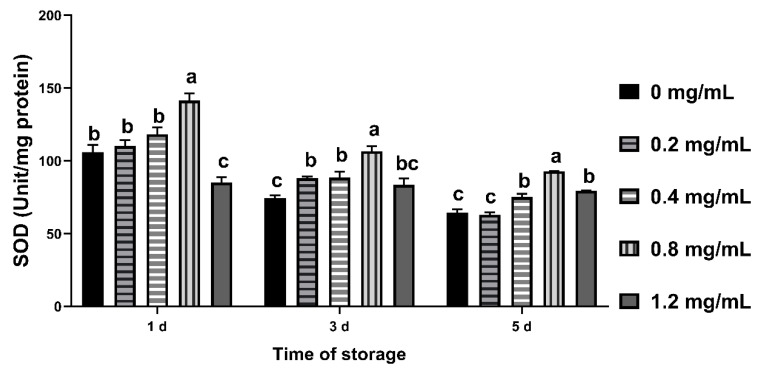
Effects of CGA with different concentrations on SOD activity of chilled ram sperm. Note: Different letters above the column indicate significant difference, the same or containing the same letter indicate insignificant difference.

The SOD activity of 0.4 mg/mL and 0.8 mg/mL groups was higher than control group from day 3 to 5 (*p* < 0.05), and the value of 0.8 mg/mL group was higher than other groups at different time points (*p* < 0.05, Figure 5).

### 3.8. Effects of CGA on ATP Content

**Figure 6:**
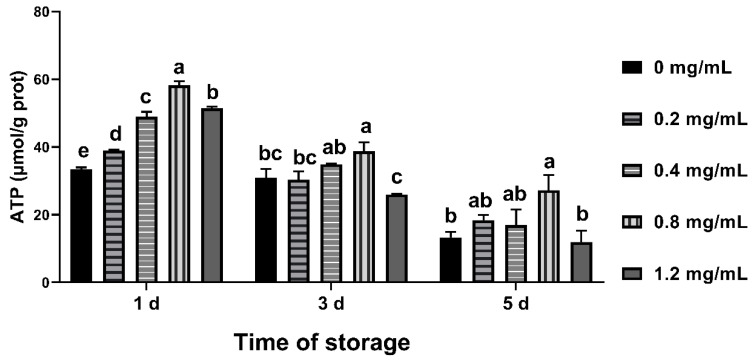
Effects of CGA with different concentrations on ATP content of chilled ram sperm. Note: Different letters above the column indicate significant difference, the same or containing the same letter indicate insignificant difference.

On the 1st day of semen preservation, ATP concentration in all CGA supplementation groups was higher than the control group (*p* < 0.05). The ATP concentration of 0.8 mg/mL group was the highest from day 1 to 5 (Figure 6).

## 4. Discussion

When the balance of ROS production and elimination was disrupted, excessive ROS caused damage to sperm [22,34]. ROS can not only lead to DNA chain break or sister chromatid exchange, but also oxidize key enzymes in the methylation process, leading to DNA methylation [35,36]. In addition, lipid peroxidation can also damage sperm. Experiments have proved that adding antioxidants to the semen extender of goats [37], cattle [38], pigs [39] and horses [40] can effectively improve sperm motility and eventually improve the effect of artificial insemination [41], which is of great significance for the development of animal husbandry.

In sperm kinematic parameters, VCL, VSL and VAP are positively correlated with sperm motility, whereas VCL is highly correlated with sperm fertilization ability [42]. A factor that may contribute to the decline of sperm kinetics (VCL, VSL and VAP) is its density. In addition, higher values for VCL and VAP were measured in extenders containing low-density lipoprotein, compared with extender egg yolk [43]. These results may be helpful to explain the lower sperm velocity in current experiment.

The current experiment indicated that CGA could significantly reduce the content of ROS and MDA during low temperature storage of semen, which was consistent with Pereira’s research results on the effect of CGA on pig semen [25]. The content of MDA in sperm increased with the prolongation of semen chilled storage in vitro. This is consistent with the discovery of the harmful effects of liquid storage on the level of MDA [44]. In vivo and in vitro studies have verified that CGA can chelate with aluminum ions and reduce lipid peroxidation and oxidative stress damage caused by aluminum ions [45].

In this experiment, with the prolongation of sperm preservation time, the integrity of sperm plasma membrane decreased significantly, which may be concerned in the destruction of sperm plasma membrane and protein distribution by ROS [46]. The sensitivity of sperm to cryoprotectants and the difference of compound concentration may affect the functional integrity of sperm. In the present study, 0.8 mg/mL CGA significantly maintained high plasma membrane integrity and acrosome integrity of chilled ram sperm. It has also been reported that the difference in the number and type of phospholipids between sperm types and regions could intervene in the stability of sperm membranes during low temperature storage [47]. In present study, the plasma membrane integrity of CGA supplementation groups was higher than control group, which is consistent with the result of Namula’s research on the effect of chlorogenic acid on semen quality of freeze–thaw pigs [27].

CAT and SOD are antioxidant enzymes widely existing in sperm cells. SOD can transform O^2−^ into H_2_O_2_ through disproportionation reaction, and H_2_O_2_ is converted into H_2_O through CAT to eliminate the influence of ROS. In the current study, CAT activity of 0.2–0.8 mg/mL CGA groups was higher on the 1st day, and that of 0.8 mg/mL group was higher on the 5th day, which indicated that 0.8 mg/mL CGA mitigated oxidative stress. It was found that the activity of SOD in chilled semen was positively correlated with sperm motility [48]. In this study, the value of SOD activity in 0.8 mg/mL CGA group was higher than control group at different time points, which indicates that 0.8mg/mL CGA has a positive effect on SOD activity. Treatment of ram sperm with BSA conjugated to OA has been reported to result in increased SOD activity after 48 h of storage at low temperature [12]. The results of this study are consistent with it.

Sperm requires ATP produced from the middle of the mitochondria to main motility [49]. However, due to species, its production and metabolism process is not fully understood. Some researchers have found correlation between the fertility in different bull breeds [50] and the motility in starlet [51], whereas other reports on mammals have found that there is no correlation between fertility and ATP content of sperm [52,53], but it is related to the motility parameters [52]. In the present study, the ATP concentration in 0.8 mg/mL group was higher than that of other groups on 5th day of semen preservation, which was corresponding to viability and PM during semen preservation and consistent with stallion [51]. With the prolongation of semen preservation time, sperm ATP concentration decreased gradually, which was corresponding to viability and PM.

High concentrations of antioxidants may damage the functional integrity of acrosome and membrane of sperm [54,55]. In the present study, the chilled ram sperm plasma membrane and acrosomal integrity in 1.2 mg/mL group was lower than 0.8 mg/mL group on the 7th day of semen preservation, possibly due to the increase in cell membrane permeability, which resulted in decreased sperm quality.

## 5. Conclusions

In conclusion, the ram semen extender containing CGA improved chilled ram sperm kinematic parameters, plasma membrane integrity, acrosome integrity, total antioxidant capacity, CAT, SOD activity, decreased the accumulation of ROS and MDA, and contained the ATP content. The optimum CGA concentration in the semen extender was determined to be 0.8 mg/mL. The reproductive potential of chilled ram sperm needs further study.

## Figures and Tables

**Table 1:** Effects of CGA with different concentrations on kinematic parameters of chilled ram sperm.

Index	Time (d)	0 mg/mL	0.2 mg/mL	0.4 mg/mL	0.8 mg/mL	1.2 mg/mL
Viability (%)	0	91.27 ± 0.68	90.96 ± 0.48	91.67 ± 0.44	91.01 ± 0.47	92.26 ± 0.43
	1	83.53 ± 0.70 ^b^	83.08 ± 0.41 ^b^	83.74 ± 0.27 ^b^	86.52 ± 0.51 ^a^	85.24 ± 0.08 ^a^
	3	71.92 ± 0.64	74.30 ± 1.53	75.31 ± 1.13	75.13 ± 1.89	74.51 ± 0.95
	5	57.81 ± 0.06 ^c^	67.76 ± 0.49 ^b^	69.25 ± 0.38 ^a^	69.68 ± 0.76 ^a^	68.36 ± 0.10 ^ab^
	7	49.80 ± 1.08 ^c^	57.28 ± 0.9 ^b^	58.12 ± 0.36 ^b^	60.57 ± 0.51 ^a^	56.73 ± 0.46 ^b^
PM (%)	0	83.73 ± 0.65	83.09 ± 1.24	83.24 ± 0.69	82.45 ± 0.42	84.43 ± 0.64
	1	73.45 ± 0.98 ^b^	72.73 ± 0.82 ^b^	73.85 ± 0.51 b	77.78 ± 1.16 ^a^	77.14 ± 0.08 ^a^
	3	60.51 ± 0.36 ^b^	64.35 ± 1.22 ^ab^	66.28 ± 1.48 ^a^	66.08 ± 2.79 ^a^	63.01 ± 0.90 ^ab^
	5	44.76 ± 0.81 ^c^	56.96 ± 0.23 ^ab^	58.04 ± 0.03 ^a^	58.66 ± 1.13 ^a^	54.97 ± 1.02 ^b^
	7	33.92 ± 3.01 ^b^	44.17 ± 1.45 ^a^	46.08 ± 0.02 ^a^	47.75 ± 2.13 ^a^	45.62 ± 1.94 ^a^
VSL(μm/s)	0	47.80 ± 0.92	48.59 ± 0.44	50.11 ± 0.36	50.14 ± 0.69	50.14 ± 1.69
	1	33.84 ± 1.06 ^c^	38.65 ± 1.18 ^ab^	35.78 ± 1.36 ^ab^	39.54 ± 1.18 ^a^	37.39 ± 0.32 ^ab^
	3	24.60 ± 0.33	24.89 ± 0.25	24.78 ± 0.56	24.62 ± 0.12	24.29 ± 0.24
	5	30.42 ± 0.61 ^b^	27.72 ± 0.10 ^c^	30.03 ± 0.41 ^b^	32.44 ± 0.39 ^a^	29.75 ± 0.74 ^b^
	7	23.52 ± 0.20 ^b^	23.77 ± 0.49 ^b^	24.94 ± 1.11 ^ab^	26.58 ± 0.10 ^a^	23.01 ± 0.76 ^b^
VCL(μm/s)	0	82.19 ± 0.61	79.72 ± 0.64	82.62 ± 0.81	79.53 ± 0.76	79.85 ± 1.74
	1	67.13 ± 2.19 ^c^	78.29 ± 2.42 ^a^	74.06 ± 2.59 ^ab^	80.11 ± 2.27 ^a^	77.51 ± 0.71 ^a^
	3	62.19 ± 1.92	61.23 ± 0.26	60.98 ± 0.30	61.42 ± 0.94	60.21 ± 1.54
	5	66.36 ± 0.94	62.07 ± 0.27	66.19 ± 0.88	69.63 ± 0.15	66.04 ± 1.40
	7	51.18 ± 0.26 ^b^	52.56 ± 1.80 ^b^	58.56 ± 1.63 ^a^	60.98 ± 0.01 ^a^	50.06 ± 2.10 ^b^
VAP(μm/s)	0	58.12 ± 0.43	57.65 ± 0.89	58.42 ± 0.57	57.68 ± 0.93	57.29 ± 1.47
	1	47.47 ± 1.55 ^b^	55.36 ± 1.71 ^a^	52.37 ± 1.83 ^ab^	56.64 ± 1.60 ^a^	54.81 ± 0.50 ^a^
	3	43.97 ± 1.36	43.29 ± 0.18	43.12 ± 0.22	43.43 ± 0.67	42.58 ± 1.09

Note: Different letters within the same row indicate significant difference (*p* < 0.05), different letters or containing the same letter indicate no significant difference (*p* > 0.05).

**Table 2:** Effects of CGA with different concentrations on plasma membrane and acrosome integrity of chilled ram sperm.

Index (%)	Time (d)	0 mg/mL	0.2 mg/mL	0.4 mg/mL	0.8 mg/mL	1.2 mg/mL
Plasma membrane integrity	0	77.69 ± 0.40	77.72 ± 0.60	76.78 ± 0.54	76.29 ± 0.08	76.38 ± 0.73
	1	72.30 ± 0.08 ^b^	72.73 ± 0.16 ^b^	72.43 ± 0.55 ^b^	74.93 ± 0.18 ^a^	72.96 ± 0.56 ^b^
	3	60.60 ± 0.15 ^d^	63.21 ± 0.35 ^c^	65.40 ± 0.22 ^b^	69.00 ± 0.48 ^a^	68.45 ± 0.03 ^a^
	5	51.64 ± 0.16 ^d^	54.93 ± 0.19 ^c^	61.98 ± 0.38 ^b^	65.58 ± 0.55 ^a^	64.60 ± 0.54 ^a^
	7	46.72 ± 0.25 ^d^	55.21 ± 0.53 ^c^	57.50 ± 0.40 ^b^	61.15 ± 1.01 ^a^	58.07 ± 0.63 ^b^
Acrosomal integrity	0	95.55 ± 0.05	95.39 ± 0.33	95.68 ± 0.23	95.42 ± 0.43	95.00 ± 0.20
	1	79.14 ± 0.42 ^c^	81.22 ± 0.37 ^b^	82.28 ± 0.33 ^b^	84.19 ± 0.86 ^a^	78.73 ± 0.40 ^c^
	3	75.80 ± 0.73 ^b^	77.65 ± 0.40 ^b^	80.88 ± 0.53 ^a^	81.15 ± 0.89 ^a^	77.53 ± 0.24 ^b^
	5	69.15 ± 1.33 ^c^	74.09 ± 0.60 ^b^	76.09 ± 0.96 ^b^	80.61 ± 1.49 ^a^	75.30 ± 0.46 ^b^
	7	57.57 ± 2.31 ^c^	61.62 ± 1.42 ^c^	67.60 ± 0.86 ^b^	75.88 ± 0.58 ^a^	67.60 ± 0.75 ^b^

Note: Different letters within the same row indicate significant difference (*p* < 0.05), different letters or containing the same letter indicate no significant difference (*p* > 0.05).

## Data Availability

The data will be made available upon reasonable request.
